# Neurofeedback treatment for Attention Deficit Hyperactivity Disorder in adults

**DOI:** 10.1192/j.eurpsy.2024.1288

**Published:** 2024-08-27

**Authors:** B. Fernández, J. Barros, E. Machado, E. Maldonado, A. R. Moreira, H. J. Gomes

**Affiliations:** Psychiatry, ULSNE, Bragança, Portugal

## Abstract

**Introduction:**

Attention-deficit/hyperactivity disorder (ADHD) is one of the most prevalent neurodevelopment disorders in the world. Clinical guidelines for ADHD recommend multimodal treatment approaches, with current evidence suggesting that medication, including methylphenidate and various amphetamine formulations, in conjunction with psychosocial treatment are most effective in the short-term and long term. Over the last decade, an increasing number of studies investigating non-pharmacological treatments have been published, such as cognitive therapy, Neurofeedback (NF), Transcranial direct current stimulation with the aim of treating ADHD patients.

**Objectives:**

We comprehensively reviewed literature searching for studies on the effectiveness and specificity of NF for the treatment of ADHD. The aim of this review is to understand if there is scientific evidence in using of electroencephalogram **(**EEG)-Neurofeedback for treating patients with ADHD.

**Methods:**

We did a non systemathic review using pubmed and google schoolar databases in order to analyze the influence and effects of therapy in patients diagnosed with ADHD and under treatment based on EEG Neurofeedback. We analyzed 18 systhematic reviews and metha-analysis and 2 case control studies.

**Results:**

Accourding to the systhematic reviews results showed positive and significant effects in the visual memory, attention and visual recognition (spatial working memory). EEG also showed improvement in upper alpha activity in a resting state (open-eyed) measured from the occipital area, which similarly indicated improvement in the cognitive domain (attention). Compared to non-active control treatments, NF appears to have more durable treatment effects, for at least 6 months following treatment.

**Conclusions:**

In conclusion, it is possible to affirm that a neuromodulating effect of the therapy positively influences cognitive processes, mood, and anxiety levels in patients with ADHD and is associated with significant long-term reduction in symptoms. Though limitations exist regarding conclusions about the specific effects of NF, the review documents improvements in school, social, and family environments. However, future efforts should focus on implementing standard neurofeedback protocols, ensuring learning, and optimizing clinically relevant transfer and more studies are needed for a properly powered comparison of follow-up effects between NF and active treatments and to further control for non-specific effects.

**Disclosure of Interest:**

None Declared

